# Traumatic brain injury-induced cerebral microbleeds in the elderly

**DOI:** 10.1007/s11357-020-00280-3

**Published:** 2020-10-03

**Authors:** Luca Toth, Andras Czigler, Peter Horvath, Balint Kornyei, Nikolett Szarka, Attila Schwarcz, Zoltan Ungvari, Andras Buki, Peter Toth

**Affiliations:** 1 Department of Neurosurgery, University of Pecs, Medical School, 2 Ret Street, Pecs, 7624 Hungary; 2 Institute for Translational Medicine, University of Pecs, Medical School, Pecs, Hungary; 3 Department of Radiology, University of Pecs, Medical School, Pecs, Hungary; 4 Reynolds Oklahoma Center on Aging, Department of Biochemistry, University of Oklahoma Health Sciences Center, Oklahoma City, OK USA; 5 Department of Public Health, Semmelweis University, Faculty of Medicine, Budapest, Hungary; 6 MTA-PTE Clinical Neuroscience MR Research Group, Pecs, Hungary

**Keywords:** Microbleed, Cerebral microhaemorrhage, Brain trauma, Mild traumatic brain injury, Ageing, Vascular changes

## Abstract

Traumatic brain injury (TBI) was shown to lead to the development of cerebral microbleeds (CMBs), which are associated with long term cognitive decline and gait disturbances in patients. The elderly is one of the most vulnerable parts of the population to suffer TBI. Importantly, ageing is known to exacerbate microvascular fragility and to promote the formation of CMBs. In this overview, the effect of ageing is discussed on the development and characteristics of TBI-related CMBs, with special emphasis on CMBs associated with mild TBI. Four cases of TBI-related CMBs are described to illustrate the concept that ageing exacerbates the deleterious microvascular effects of TBI and that similar brain trauma may induce more CMBs in old patients than in young ones. Recommendations are made for future prospective studies to establish the mechanistic effects of ageing on the formation of CMBs after TBI, and to determine long-term consequences of CMBs on clinically relevant outcome measures including cognitive performance, gait and balance function.

## Introduction

Traumatic brain injury (TBI) is a serious health problem worldwide [1, 2]. In addition to its acute clinical significance, TBI was shown to lead to chronic neurological dysfunction (including long-term impairment of gait and cognition) and to promote psychiatric disorders [3, 4]. After the direct neuronal damage caused by the impact, a divergent process is initiated resulting in secondary injury of neuroglial tissue [2, 3, 5–9]. Injury of cerebral vessels and cerebrovascular dysfunction play a central role in the pathological processes of secondary injury [7, 8, 10, 11]. After mild, moderate and severe TBI mitochondrial dysfunction, oxidative stress and redox-dependent activation of matrix metalloproteinases (MMP) are enhanced, contributing to the damage of the microvascular wall and to the development of blood-brain barrier (BBB) dysfunction [2, 6, 10, 12–15]. These pathological processes contribute to the formation of microhaemorrhages around brain microvessels [7, 16, 17]. Cerebral microhaemorrhages, also referred as cerebral microbleeds (CMBs), are small hemosiderin deposits (less than 5 to 10 mm in diameter) resulting from bleeding from injured small arteries, cerebral arterioles or capillaries [9, 16, 18–20]. CMBs determine clinical outcome of patients; they are associated with the development of cognitive impairment indicated by attenuated processing speed, defective attention and executive dysfunction [21–24]. They also promote psychiatric disorders such as major depressive episodes [3, 4, 9, 16, 22]. Presence of CMBs is linked to dysfunction in gait coordination and balance: shorter stride length and decreased general functionality [25–28].

The elderly population is prone to suffer TBI [1]. The most frequent cause of trauma among the elderly is unintentional fall [1, 29], usually due to orthostathic hypotension and dehydration. The possibility of falls is exacerbated by impaired balance due to decreased muscular strength and different types of neuropathies [1, 30]. The prognosis of older patients after TBI is worse than middle aged or young individuals [9, 29, 31] indicated by increased mortality, longer hospital stay and higher need for rehabilitation [1, 30, 31]. Rehabilitation is also less effective in the elderly than in young patients [1, 30].

There is growing evidence that ageing is an independent risk factor for the development of CMBs [16, 20, 32–34]. The number of CMBs increases approximately by 20–40% in individuals aged 65 years and older [9, 16, 18, 34]. Strong evidence suggests that CMBs are causally linked to cognitive decline and gait disturbances in the elderly [16, 17, 20, 23, 34]. There is also evidence available that various age-related vascular processes, such as oxidative stress, increased MMP activity, modification of collagen and elastin content of the cerebrovascular wall, and increased fragility of aged cerebral vessels contribute to exacerbation of CMBs in the aged brain [16, 17]. Recent data suggest that in these processes, age-related endocrine changes, especially decline in circulating level of the vasoprotective hormone insulin like growth factor 1 (IGF-1), play a central role [16, 35–37]. Importantly, the prevalence of hypertension significantly increases with age, and hypertension and ageing interact to induce cerebrovascular dysfunction and the formation of cerebral microbleeds [16, 17, 34]. Preclinical studies show that the mechanisms by which hypertension and ageing interact to increase microvascular fragility and to promote the genesis of CMBs include increased oxidative stress, vascular lipohyalinosis, induction of MMP activation and extracellular matrix remodelling [16, 17]. As mentioned above, most of these mechanisms are also induced in TBI [6, 12, 13, 15, 38]. In the following, we present 4 cases of young and aged patients with and without mild TBI and discuss the possible mechanistic interaction between ageing and TBI to induce the formation of CMBs.

## Cases

**Fig. 1:**
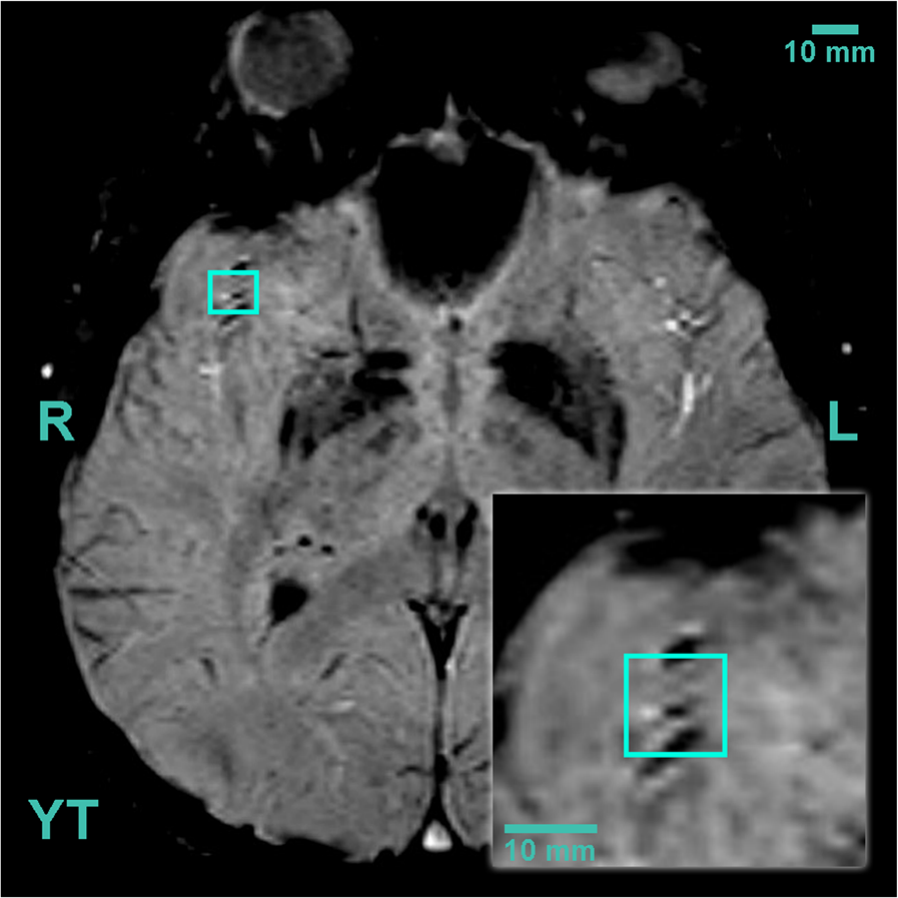
Blue square depicts a cerebral microbleed (CMB) in the right inferior longitudinal fasciculus of a young TBI patient (YT) (40-year-old male, mild TBI). On the axial susceptibility-weighted magnetic resonance image (SWI, obtained at 3 Tesla), the bleeding appears as an ovoid, hypointense lesion [20, 32, 33]

**Fig. 2:**
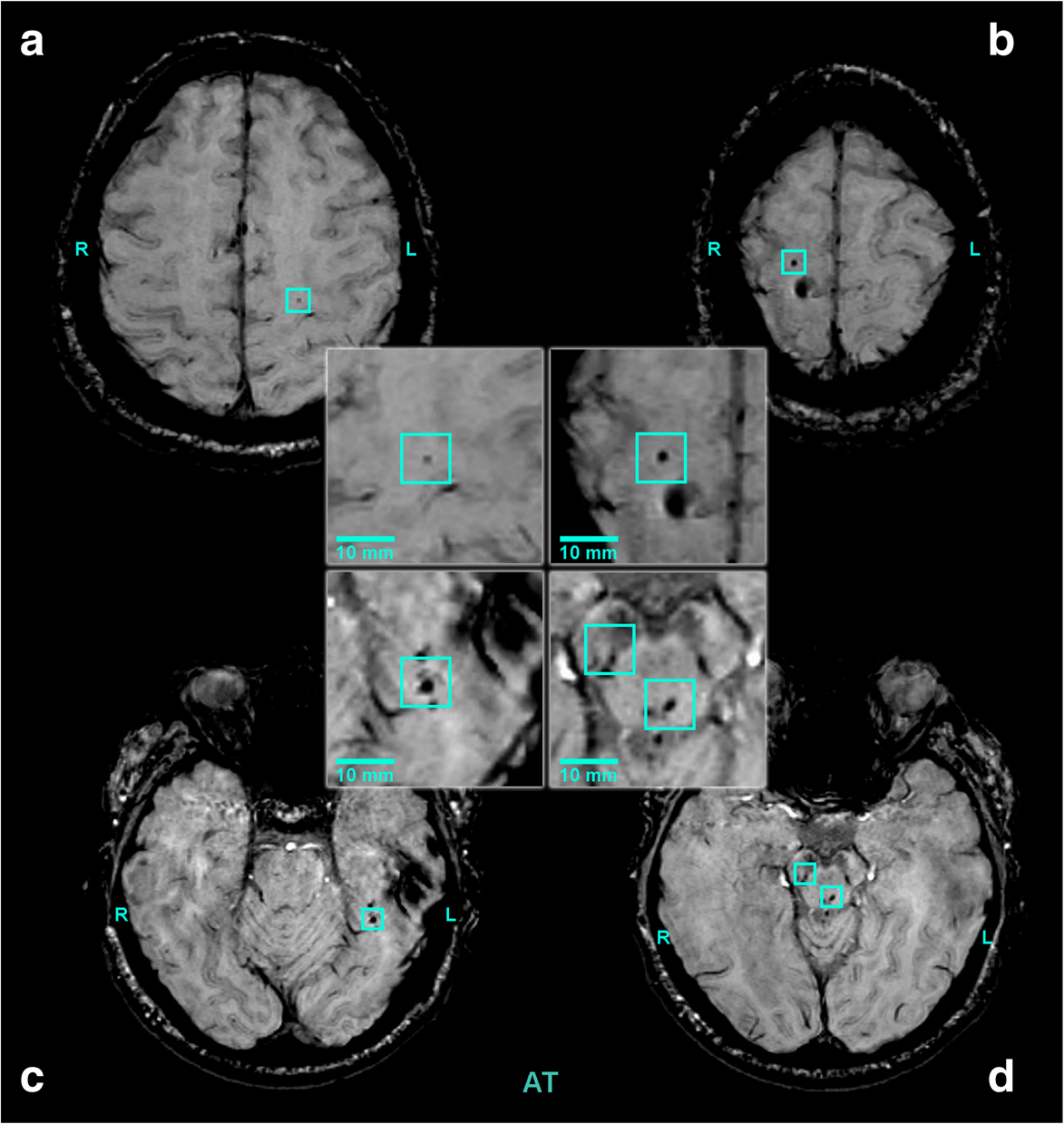
Axial susceptibility-weighted (SWI) magnetic resonance images (3 T) of an elderly patient (case 2) with mild traumatic brain injury (AT) (60-year-old, male). Cerebral microbleeds (CMBs) are highlighted by the blue boxes: A, left corona radiate; B, right corona radiate; C, left parahippocampal gyrus; D, crus cerebri, medial longitudinal fasciculus

**Fig. 3:**
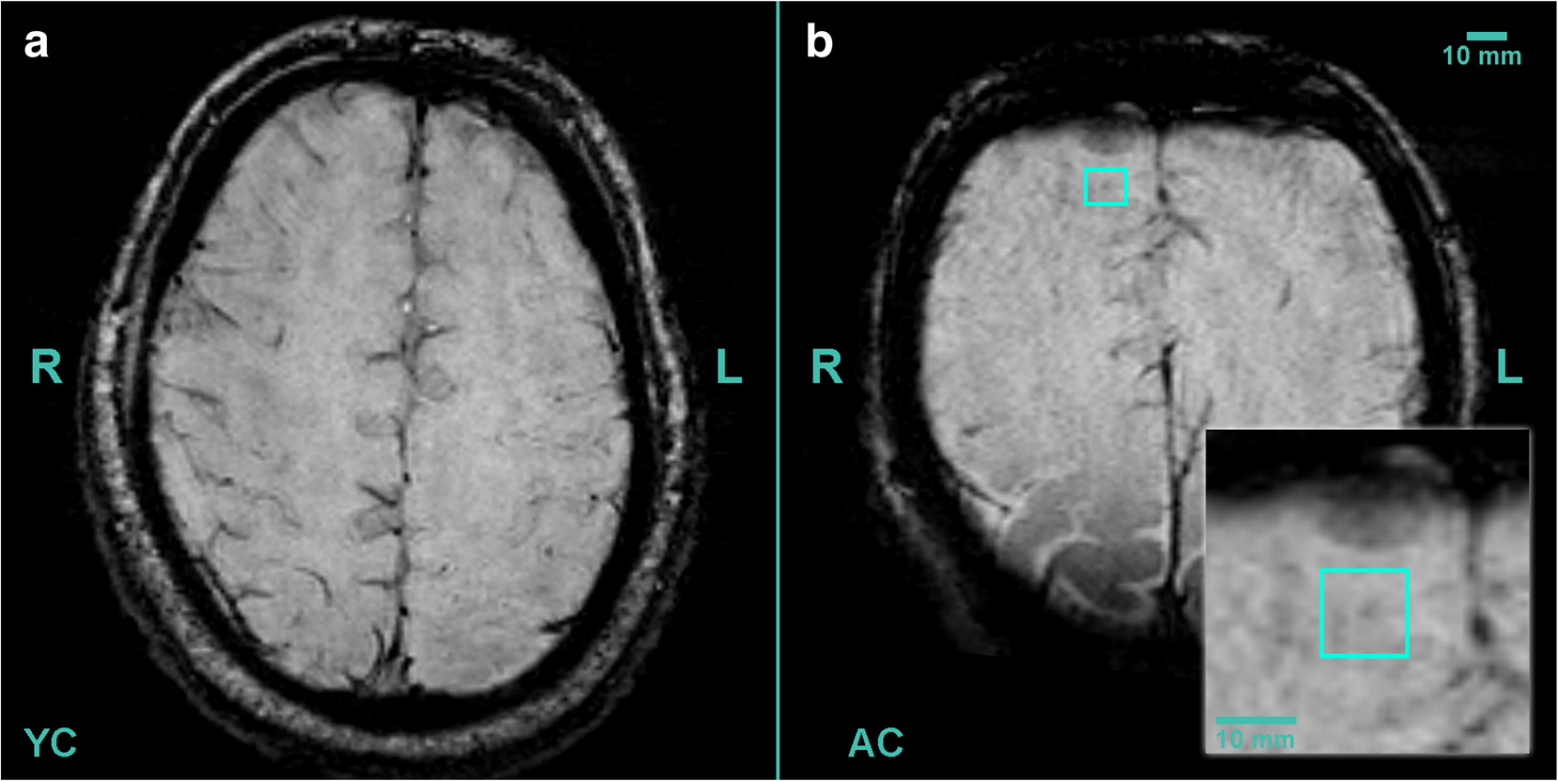
Axial (3 T) SWI MR images of aged and young control patients (without trauma). **a** Aged control (AC) patient presented one CMB lesion, located in right corona radiate, highlighted with blue square. **b** The young control (YC) patient had no cerebral lesion

This study was approved by the Regional Ethic Committee of the University of Pecs, Medical School (7270-PTE 2018). We retrospectively analysed the medical history and susceptibility weighted (SWI) MRI (Siemens Magnetom Prisma Fit 3T) series of images of two patients (40-year and 60-year-old males) who suffered mild TBI and were referred to the Department of Neurosurgery, Medical School, University of Pecs, Hungary (Table 1, Figs. 1 and 2). We also evaluated the MRI images of two patients without brain trauma (31-year and 64-year-old males) (Table 1, Fig. 3). SWI MRI was demonstrated to be more proficient to detect CMBs compared to T2* gradient echo (GRE) [19, 32]. This is due to post-processing and the augmentation of the magnetic resonance signal with signal pulse shift [19, 39, 40]. Demonstrated by SWI sequences CMBs are round- or ovoid-shaped hypointense lesions with the dimensions of 5–10 mm, encircled by cerebral parenchyma (in whole or in part) [20, 32, 40]. Exclusion criteria were the presence of any of the following: epilepsy, previous stroke, transient ischemic attack, cerebral amyloid angiopathy, chronic hypertensive encephalopathy, acute haemorrhagic leukoencephality, CADASIL, cerebral vasculitis, cerebral metastases, intracranial infections, intracranial embolism, posterior reversible encephalopathy syndrome, or any types of neurodegenerative diseases [16, 19, 20, 32]. Severity of TBI was defined by the initial Glasgow Coma Scale (GCS): mild 14–15, moderate 8–13 and severe < 8 [30]. Two independent radiologists evaluated the number and distribution of CMBs on SWI series of patients, blinded to the medical history of the cases. Location was described by the Adams Classification system and the Microbleed Anatomical Rating Scale (MARS) system [20, 41, 42].

**Table 1:** Summary of the main characteristics of the presented four patients

	Young TBI (YT)	Aged TBI (AT)	Young control (YC)	Aged control (AC)
Age at trauma	40	60	N/A	N/A
Age at MR	40	60	35	65
Sex	Male	Male	Male	Male
Cause of trauma	Traffic accident	Fall	N/A	N/A
GCS	15	15	N/A	N/A
LOC	3 min	None	N/A	N/A
PTA	None	None	N/A	N/A
Number of CMBs	2	9	0	1
Location of CMBs according to MARS	Lobar	Lobar, deep	N/A	Lobar
Adam’s grade	Grade I	Grade III	N/A	Grade I
Comorbidity	None	Hypertension	None	None

### Case 1

The 40-year-old male patient was admitted to the hospital because of a head trauma he had suffered in a road traffic accident. The initial GCS score was 15, no memory disturbances were documented, but following the accident, temporary loss of consciousness occurred for less than 3 min. Neurologic examination did not show any symptoms or signs. He had no comorbidities. CT scan showed no skull fractures or intracranial haemorrhage. On the patient’s MRI by MARS system, 2 CMBs were detected in the right temporal lobe, one was located in the cortical-subcortical border, the other one was located in the subcortical white matter (Fig. 1). According to Adam’s classification, the lesions are grade I.

### Case 2

The second patient was a 60-year-old man, who was admitted to the hospital because of a fall. GCS score was 15 at the admittance, no neurological signs or symptoms could be detected. Hypertension had been known for 20 years with dilative cardiomyopathy. CT scan showed a cerebral contusion of 7 mm in diameter in the right parietal cortex and a minor parafalcin subdural haematoma. By MRI, multiple CMBs were detected (Fig. 2). According to MARS system, 7 lobar lesions were found (2 in the right frontal lobe, 1 in the left parietal lobe, 4 in the left temporal lobe) (Fig. 2). Further 2 lesions were found in the brainstem (Fig. 2). According to the Adam’s classification, he has grade III lesions (not affecting the corpus callosum).

### Case 3

The patient was a 35-year-old man who attended the clinic with bilateral upper limb numbness. His medical anamnesis is negative for any significant pathology. Laboratory test showed no alterations. The MRI did not reveal any intracranial abnormalities, which could explain the symptoms. On the SWI images, no cerebral microbleeds were found (Fig. 3).

### Case 4

The 65-year-old male patient was presented to the clinic with back pain, without any traumatic brain injury in his history. Spondylosis was diagnosed. On the SWI images, 1 cerebral microbleed was found in left frontal lobe in the subcortical white matter (Adams’ grade I lesion) (Fig. 3).

## Cerebral microbleeds in TBI and ageing: possible mechanisms

In the presented elderly patient with mild TBI, multiple CMBs were found, which is representative to the imaging findings in patients in this age group. In the young trauma patient, the number of CMBs was markedly less, consistent with findings reported in TBI patients of this age group. It has to be noted that majority of the lesions in the older TBI patient were located in typical brain areas for traumatic CMBs (corona radiata, longitudinal fasciculus) (Fig. 2); however, two lesions were detected in the brainstem, an atypical location for traumatic microbleeds [3, 19, 23, 25, 34, 43]. Cerebral microbleeds in deep cerebral areas are thought to be due to cerebral angiopathy induced by hypertension [33, 43]. The presented cases support the hypothesis that ageing and TBI may interact to promote the development of CMBs.

**Fig. 4:**
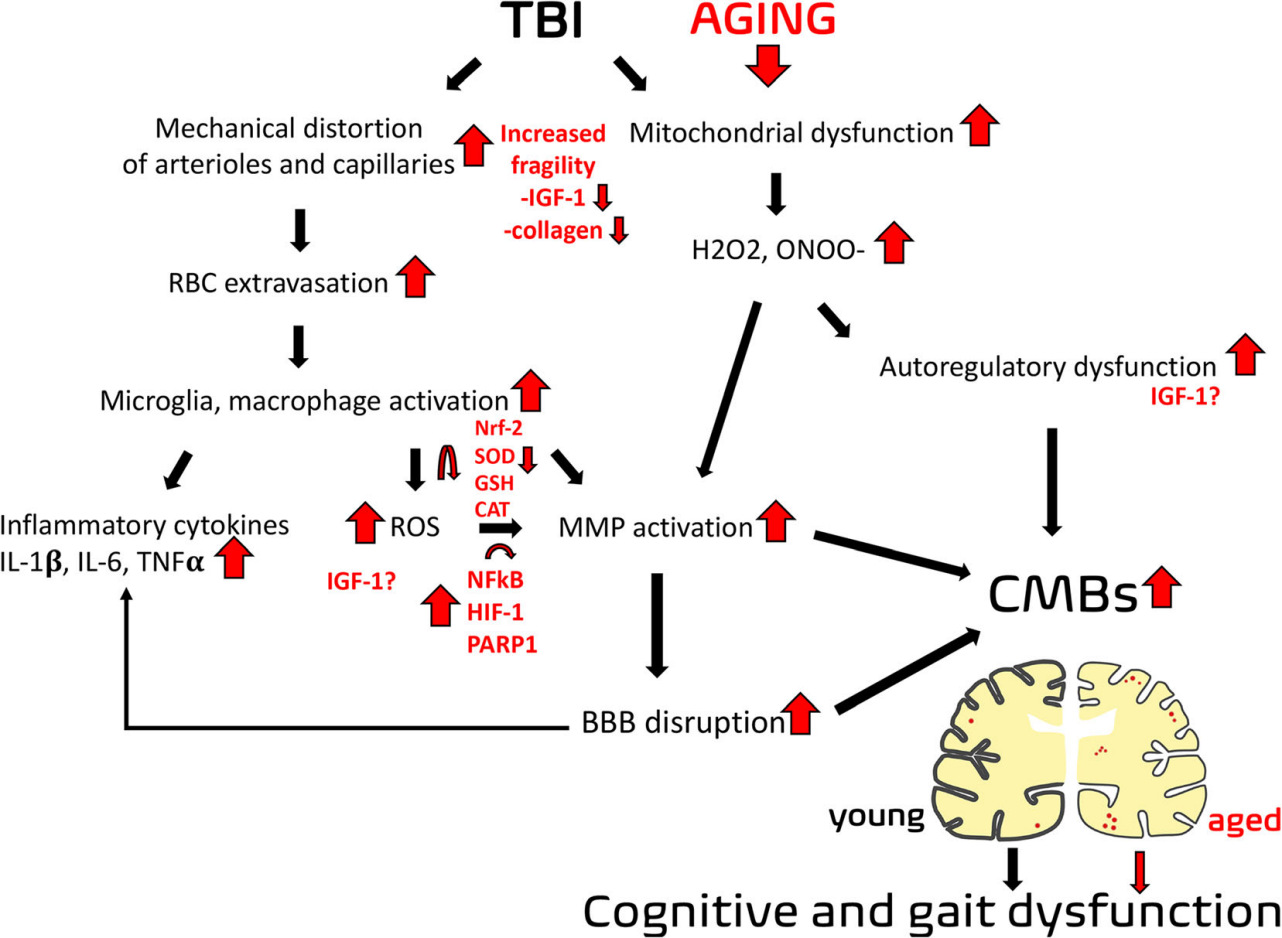
Possible mechanisms of the synergistic effect of traumatic brain injury and ageing on the formation of cerebral microbleeds. Please see detailed description in the text. Traumatic brain injury (TBI) leads to mechanical distortion of cerebral vessels, which may directly lead to injury of the vascular wall and formation of microhemorrhages around cerebral arterioles and capillaries. This mechanism may be enhanced by ageing via age-related changes of the cerebrovascular wall leading to increased fragility of the vessels. TBI-induced mitochondrial oxidative stress and production of reactive oxygen species (ROS) and inflammatory mediators in activated microglia and macrophages following TBI may be exacerbated by ageing due to the age-related decreased antioxidant cellular mechanisms. In addition to the direct damage of the cerebrovascular wall, TBI-induced autoregulatory dysfunction may contribute to the development of cerebral microbleeds by placing increased hydrostatic burden on the cerebral microcirculation due to lack of proximal protection against blood pressure. Autoregulatory dysfunction may be exacerbated by age-related deficiency of circulating insulin-like growth factor 1 (IGF-1). These mechanisms converge on the disruption of the blood-brain barrier (BBB) and formation of cerebral microbleeds and consequent cognitive and gait dysfunction following TBI. We posit that enhanced vascular fragility, increased cerebrovascular oxidative stress and autoregulatory dysfunction in the elderly result in the formation of more cerebral microbleeds and more severe impairment of cognitive and gait function compared to young patients

In the following section, the possible mechanisms by which ageing promotes TBI-induced CMBs and exacerbates CMB-related neuronal dysfunction are discussed (Fig. 4). Sudden accelerating and decelerating shearing forces during head trauma likely play a central role in the development of CMBs, which accompanies TBI-related diffuse axonal injury [7, 9, 25, 44]. Mechanical distortion of endothelial cells leads to disruption of the BBB and capillary damage, provoking blood extravasation and the formation of small haemorrhagic lesions [8, 44–46]. Traumatic microbleeds are characteristic in the vicinity of small cerebral arteries, arterioles, capillaries and bridging veins [8, 44, 46]. Collagen (mainly I and III) plays an important role in vascular stiffness and tissue repair [16, 36, 47]. During ageing, vascular collagen is modified due to age-related mineralisation [16, 36]. The mineralised and modified collagen is more fragile; thus, the aged vessels are more susceptible to be injured after trauma [16, 48–50]. In addition to the increased vascular stiffness due to age-related enhanced collagen content of the cerebrovascular wall, ageing promotes the structural modification of elastin leading to impaired elasticity of the vessels [16, 36, 47–49]. These age-related changes in biomechanical properties of cerebral vessels most likely exacerbate the abovementioned TBI-related mechanical injury. Interestingly, in animal models, both mild and severe TBIs were shown to lead to a decrease in cerebrovascular stiffness indicated by attenuated modulus of rigidity, as well as an increase in the radius of the vessels in the affected cerebral tissue. This potentially contributes to reactive local hyperperfusion [51–54]. One can hypothesise that this hydrostatic burden may exacerbate TBI-related vascular injury in the elderly. After cerebral vessels become leaky, extravasated erythrocytes and plasma triggers activation of microglia and macrophages, migration of neutrophils and increased production of cytokines [7–9, 55]. This inflammatory reaction contributes to neuronal damage and dysfunction as shown by demyelination, loss of neuropil, impaired fluid removal in perivascular spaces, impaired neurogenesis and differentiation [7, 33, 45, 55, 56]. These are most likely exacerbated in ageing, as the number of activated microglia is increased in the aged brain, being responsible for excessive and prolonged expression of inflammatory cytokines IL-1, IL-6, IL-12 and TNF α [56–58].

TBI induces mitochondrial dysfunction and excessive production of mitochondria-derived free radicals (mostly hydrogen peroxide and peroxynitrite), which are further exacerbated by accumulation of hemosiderin, heme and free iron in the cerebral parenchyma and in endothelial cells [7, 45, 55, 59]. This results in further BBB disruption and formation of vasogenic and cytotoxic oedema, leading to a vicious cycle [7, 8, 10, 45]. The aforementioned cascade is thought to be more critical in the elderly. For example, in ageing, TBI-induced microglial proliferation is more pronounced than in young patients because of age-related decreased phagocytic activity, increased ROS production and enhanced leukocyte activation [11, 60, 61]. In ageing, cerebrovascular oxidative stress is increased compared to younger individuals, partly due to impaired antioxidant defence mechanisms (including dysfunction of the Nrf2-dependent cytoprotective pathways, decreased level and activity of antioxidant enzymes as superoxide dismutase (SOD), catalase and the glutathione system (GSH)) as well as up-regulation of NADPH oxidases [16, 17, 50, 58, 61–72]. One of the main sources of ROS in the cerebrovasculature are mitochondria [17, 62, 71, 73]. Mitochondrial oxidative stress has been shown to be increased after TBI as well as in ageing [2, 6, 7, 11, 12, 58, 60, 62]. Importantly, mitochondrial DNA is more prone to damage caused by reactive oxygen substances, which is also exaggerated in ageing [62, 63]. It is of note that mitochondrial oxidative stress was shown to contribute to autoregulatory dysfunction following TBI, which may result in downstream injury of the cerebral microcirculation due to pressure and volume overload [10, 12]. This hydrostatic burden contributes to the development of both BBB disruption and formation of microhaemorrhages [7, 10, 15, 16]. This pathophysiological mechanism is likely enhanced by ageing and should be studied in the future [35].

Matrix metalloproteinases (MMPs) play a central role in structural microvascular damage and BBB disruption after TBI [10, 13–15]. Importantly, ageing results in increased MMP activity in the brain [74]. MMPs are activated by age-related crosslinking of vascular collagen and are induced by age-related oxidative stress and by decreased activity of protease inhibitors [16, 17, 36, 48, 49, 74]. TBI can induce MMP activity via activating transcription factors such as hypoxia-inducible factor 1 alpha (HIF1α), NF-kB and poly(ADP-ribose) polymerase-1 (PARP-1) [7, 11, 49, 73]. These transcription factors are found to be induced in the elderly [17, 61]. Age-related activation of NF-kB and HIF1 α alters mitochondrial and cellular repair function, as well, augmenting inflammatory mechanisms and further potentiating TBI-induced secondary injury [61].

It is important to note that age-related endocrine changes, specifically, the age-related decline in IGF-1 may play a central role in the development of age-related, hypertension-induced formation of microbleeds [35, 75, 76]. It was recently demonstrated that deficiency of circulating IGF-1 after genetic knock-down of hepatic production of the hormone in mice exacerbates the formation of cerebral microbleeds in response to hypertension, mimicking the ageing phenotype [35, 76]. IGF-1 is also known to confer multifaceted neuroprotective effects [75]. Important in this regard is that the GH/IGF-1 axis is the most sensitive to be impaired following TBI [77, 78]. Shearing forces acting on the hypothalamo-hypopituitary system, TBI-related increased intracranial pressure, haemorrhage and oedema formation and consequent local circulatory deficit have been suggested to contribute to the impairment of the GH/IGF-1, which affects approximately 10–20% of TBI patients [77, 79, 80]. GH/IGF-1 deficiency can last for years following TBI and has been suggested to significantly contribute to chronic cognitive decline, as well as to decrease quality of life of TBI patients [75, 77, 81, 82]. It is logical to posit that TBI-related attenuation of IGF-1 production may be exacerbated in aged subjects. Future studies should determine the contribution of age-related IGF-1 deficiency to the genesis of CMBs and/or exacerbated CMB-induced neuronal damage in older adults.

## Clinical importance

### Gait dysfunction

Posture and gait necessitate coordinated operation of cortical (motor cortex) and subcortical areas (basal ganglia, thalamus, cerebellum, the limbic system, midbrain, pons, medulla and spinal locomotor network) [16, 83]. Development of CMBs results in gait dysfunction by damaging these centres and disrupting the communicating pathways between them [27, 28, 58]. Accordingly, CMBs in temporal and frontal lobes, in basal ganglia and corona radiata (independent of white matter lesions) showed a significant correlation with poor gait function in elderly patients [20, 27]. In the elderly, gait disturbance manifests as impaired stride length, double support time, cadence and decreased performance on the timed up and go tests [27, 28, 84]. Interestingly, only one case series investigated the effect of traumatic microbleeds on gait dysfunction [85]. This study showed that SWI positive TBI patients developed vestibular or balance abnormalities [85]. It is logical to postulate that TBI exacerbates gait dysfunction in the elderly, and gait disturbance of the elderly most likely is a central factor in the increased incidence of TBI amongst them due to increased propensity to fall. Future clinical studies are evidently needed to clarify the possible interactions between ageing and TBI on gait function and the possible role of cerebral microbleeds.

### Cognitive dysfunction

The association between the number and distribution of CMBs of different etiologies and cognitive decline has been widely analysed; however, the underlying mechanism is not fully understood. It is proposed that in development of cognitive decline, cumulative effects of the lesions as well as damage in specific anatomical locations are critical [23, 24, 33, 65]. For example, microstructural damage of fronto-subcortical circuits linking prefrontal areas to basal ganglia is associated with impairment in executive function of healthy individuals in all age groups of patients with vascular disease, whereas disarrangement of pathways from the mentioned areas projecting to thalamus results in memory disturbances [23, 24, 33, 86, 87].

In non-demented healthy elderly patients, presence of deep, subcortical CMBs was related to deterioration of global cognitive performance, particularly affecting executive function, memory and information processing, while strictly lobar CMBs resulted in executive dysfunction, decreased processing speed and gait disturbances, as well [21, 33, 83, 88].

Despite the aforementioned association between brain trauma and the development of microbleeds, limited information is available regarding the effect of traumatic CMBs on cognitive outcome [3, 9, 44]. A single case study proposed a connection between traumatic CMBs following mild TBI and decline in cognitive performance of a previously healthy 57-year-old male patient [5]. This was further substantiated by studies showing that in mild TBI patients’ number of traumatic CMBs correlated with altered neurocognitive function, impaired short-term memory, concentration difficulties and depression [3, 4, 44, 87]. Interestingly, the number of lesions in the acute stage predicted the progress of post-concussion syndrome and decline in processing speed a year after the injury [87]. Although it seems logical to posit that (even mild) brain trauma results in enhanced cognitive disturbances in elderly individuals, to our best knowledge, no studies have tested this hypothesis. Thus, future clinical research should investigate the synergistic effect of ageing and TBI-related formation cerebral microbleeds on cognitive decline.

## Conclusion and perspective

Traumatic brain injury in older adults is associated with the development of multiple CMBs (Fig. 2); however, our case report does not provide statistically relevant data to support the synergistic effect of ageing and TBI on the formation of cerebral microbleeds after brain trauma. Future clinical studies should determine the predictive value of CMBs in older TBI patients in order to estimate long-term outcome, including detailed characterization of their effect on gait and cognitive function.

Specific cellular and molecular mechanisms should be identified that could be targeted pharmacologically to prevent the development of CMBs and/or limit their deleterious effects on neuronal survival and function in the elderly after brain trauma. Testing the role of different factors involved in the synergistic pathways between ageing and TBI in formation of microbleeds could be tested by applying brain trauma of different severity on preclinical models of accelerated vascular ageing. For example, the specific role of IGF-1 deficiency in the development of traumatic microbleeds could be tested by studying the development of cerebral microhemorrhages after brain trauma in mice with viral knockdown of hepatic production of IGF-1, and the protective effect of IGF-1 supplementation could be tested [76]. As outlined above, age-related increased oxidative stress is a likely factor enhancing the formation of microbleeds following TBI. In this regard, age-dependent decreased antioxidant mechanisms play a pivotal role. Thus, it would be logical to study the formation and characterize TBI-induced CMBs in the previously used Nrf2-deficient mice [68]. Applying a similar theoretical approach, TBI-induced formation of cerebral microbleeds should also be studied in mice overexpressing mitochondrial catalase, which was previously demonstrated to effectively attenuate cerebrovascular mitochondrial oxidative stress [72, 73]. Various pharmacological interventions have been shown to decrease cerebrovascular oxidative stress and its consequences. For example, hypertension- and ageing-induced development of microhaemorrhages was prevented by treatment the animals with resveratrol, and the mitochondrial antioxidant peptide SS-31 [17, 67]. The potential positive effect of these compounds should be tested on the formation and development of TBI-induced cerebral microbleeds in ageing. Based on recent results showing that restoring cellular NAD^+^ levels in aged mice by treatment with nicotinamide mononucleotide (NMN), a key NAD^+^ intermediate, rescues neurovascular function, increases cerebral blood flow, and improves performance on cognitive tasks, we posit that NMN treatment likely prevent the formation of TBI-induced formation of cerebral microbleeds in both young and aged laboratory animals, as well [66]. Future studies should verify the preventive/protective effects of dietary intake of the NAD^+^ boosting compounds quercetin and luteolin in patients after TBI [89]. Other possible neuroprotective pathways should be studied, as well. For example, neurotrophins, such as brain-derived neurotrophic factor (BDNF) acting on tropomyosin receptor kinase B (TrK/B) receptors, have a significant role in neuronal survival, synaptic plasticity and neurogenesis under various pathological conditions [90–94]. Following TBI, the level of BDNF is temporarily increased to exert neuroprotection [91–93]. Interestingly, the level of BDNF significantly decrease with age, and it is also attenuated in chronic cardiac failure being associated with ageing, as well [90, 93–97]. Therefore, ageing presumably limits the protective increase in BDNF after TBI. This hypothesis and the role of BDNF in age- and TBI-related neuronal dysfunction should be tested in the future.

## Data Availability

The manuscript has not been published previously.
